# Zero tolerance for 0%? How should clinicians and other practitioners respond to the use of alcohol‐free and low‐alcohol products in higher risk groups

**DOI:** 10.1111/add.70244

**Published:** 2025-11-09

**Authors:** John Holmes, Christopher K. Oldroyd, Colin Drummond, Matt Field, Inge Kersbergen, Michael E. D. Allison

**Affiliations:** ^1^ School of Medicine and Population Health University of Sheffield Sheffield UK; ^2^ National Institute for Health and Care Research (NIHR) Cambridge Biomedical Research Centre Cambridge UK; ^3^ Cambridge Liver Unit Cambridge University Hospitals NHS Foundation Trust Cambridge UK; ^4^ National Addiction Centre, Institute of Psychiatry, Psychology and Neuroscience King's College London London UK; ^5^ School of Psychology University of Sheffield, Interdisciplinary Centre of the Social Sciences (ICOSS) Building Sheffield UK

**Keywords:** alcohol dependence, alcohol policy, clinical guidelines, harmful drinking, hazardous drinking, liver cirrhosis, non‐alcoholic, treatment guidelines, zero alcohol

## Abstract

Alcohol‐free and low‐alcohol drinks (no/lo drinks) are now widely available and popular with consumers in high‐income countries; however, it is unclear whether clinicians and others working to prevent or treat severe alcohol‐related health problems should take a zero‐tolerance approach to these alcohol‐like products or encourage patients to try them. We argue that no/lo drinks may have an important role to play for people who drink at high‐risk levels and those with alcohol use disorders (AUD) or alcohol‐related liver disease (ARLD), particularly where debate and guidance related to treatment of these problems considers goals other than abstinence. The limited available evidence available suggests no/lo drinks may be useful in supporting attempts to reduce alcohol consumption or maintain abstinence among high‐risk drinkers who do not have severe AUD or ARLD; however, they may also entail significant risks of relapse in those recovering from AUD. We therefore need further experimental and longitudinal studies testing whether use of no/lo drinks can lead to, or support, reductions in alcohol consumption. We particularly need high‐quality experimental studies to test whether exposure to and sustained use of no/lo drinks affects treatment and recovery outcomes. Evidence is also needed on which subgroups of AUD and ARLD patients would benefit or be at risk from use of either alcohol‐free or low‐alcohol drinks. Finally, guidance should recognise that many patients already use these products and that a zero‐tolerance approach may alienate patients or erode trust in clinicians.

## INTRODUCTION

Alcohol‐free and low‐alcohol (no/lo) drinks are increasingly available and popular with consumers in high‐income countries. This has sparked interest from health professionals regarding the potential benefits and risks these products present for tackling alcohol‐related harm. Scientific research and policy debate has so far focused on the general population and public health perspectives [1, 2, 3]. It has paid less attention to the use of no/lo drinks by those at greatest risk from alcohol [4, 5]. This includes those consuming at high‐risk levels, those with or recovering from alcohol use disorder (AUD), or those with alcohol‐related health conditions, particularly alcohol‐related liver disease (ARLD). Clinicians and other treatment providers are increasingly likely to encounter such people who are already consuming no/lo drinks or are interested in doing so to control or reduce their alcohol consumption. However, it is unclear whether they should take a zero‐tolerance approach to patients using these alcohol‐like products or support them in doing so. This article therefore examines the potential role of no/lo products and the challenges related to their use among higher risk groups.

We largely consider alcohol‐free and low‐alcohol drinks as a single category, as there is little evidence available to inform separate discussion of each product type. However, we do note some instances where the difference may matter. Although the definitions used in the cited studies vary, we generally use terminology in line with the standard UK definitions: ‘alcohol‐free’ products are those containing only trace amounts of alcohol (i.e. ≤0.05% alcohol‐by‐volume, ABV); ‘low‐alcohol’ products are those containing 0.05%–1.2% ABV; and no/lo drinks represent these categories combined. Approximately 80% of UK no/lo drinks sales are of alcohol‐free products [6].

## ALTERNATIVE END GOALS FOR ALCOHOL‐RELATED TREATMENT

Treatment of AUD and ARLD traditionally emphasises abstinence as the clinically preferred goal and outcome. Indeed, until recently, the clinical consensus for the treatment of AUD was that a return to sustained moderate or controlled drinking becomes unlikely for people with increasingly severe alcohol dependence [7, 8]. Mutual aid organisations, such as Alcoholics Anonymous, have gone further in viewing moderation as anathema to recovery. Similarly, complete abstinence remains the primary treatment goal for ARLD patients and is embedded in every major guideline on management of the condition; not least because it improves clinical outcomes at all stages of disease [9].

In reality, patients with severe AUD or ARLD do not routinely achieve abstinence. For example, only around half of patients with alcohol‐related liver cirrhosis or severe alcohol‐associated hepatitis stop drinking altogether [10, 11]. Among people with AUD who receive treatment, moderation is often a more likely long‐term recovery outcome than complete abstinence [12, 13]. Furthermore, making abstinence a requirement for receiving care may discourage people with AUD from seeking and benefiting from treatment for a condition with the highest treatment gap of any mental disorder [12, 14]. There is therefore increasing interest in moderation or harm reduction as alternative treatment and recovery goals [15, 16].

Although moderation or harm reduction approaches remain controversial [14], there is some evidence that people with AUD who would otherwise have been denied access to treatment may achieve some health benefits from moderation [12]. Organisations including the UK National Institute for Health and Care Excellence (NICE) and the World Health Organization now recommend non‐abstinence goals in their clinical guidelines for treating AUD [17, 18], while the US Food and Drug Administration and the European Medicines Agency consider primary outcomes short of complete abstinence as legitimate outcomes in phase 3 pharmacotherapy trials related to AUD [19, 20]. Supporting moderation is also the principal benefit of widely recommended relapse prevention medicines, such as naltrexone, secondary prevention initiatives targeting at‐risk drinkers, such as brief interventions [21], and managed alcohol programmes, which are increasingly used with people who are experiencing homelessness and have AUD or other substance use disorders in North America [22]. In this context, no/lo drinks may have potential to support patients to achieve moderation, either as an end goal or as a precursor to abstinence. However, the evidence to date suggests this may not be free from risk.

## POTENTIAL BENEFITS AND RISKS OF NO/LO DRINKS

Much of the evidence on no/lo drinks relates to older, lower quality products that were marketed and sold on a smaller scale than those now available [5]. The most recent studies include natural experiments that suggest there have been small reductions in alcohol purchasing among the general population following the introduction of lower strength alcoholic drinks (e.g. beers up to 3.5% ABV) [23]. Survey research and studies of purchasing data also suggest that people consuming larger quantities of alcohol are more likely to purchase no/lo drinks than lighter drinkers [24, 25, 26], and that people increasingly use such products when attempting to reduce their alcohol consumption. However, no/lo drinks are used less often by people in lower socio‐economic groups, potentially because of their perceived higher costs, which may perpetuate alcohol‐related health inequalities if benefits mainly accrue in the least deprived [27]. More positively, qualitative research suggests people in recovery from AUD find no/lo drinks can facilitate active self‐management of their alcohol consumption, allow them to maintain supportive social relationships and negate the stigma associated with abstinence in traditional drinking contexts [28, 29]. However, others have cautioned that shared branding between no/lo and standard alcoholic drinks (e.g. Heineken 0.0 and Heineken) may mean that people with AUD are exposed to alcohol branding more often and in more settings than would otherwise have been the case, which might act as a cue to trigger relapse in some people [30].

Findings from experimental studies involving people with AUD or those who drink at high‐risk levels relate to earlier generations of no/lo products, or in some cases placebo beverages, and should therefore be interpreted with caution [5]. However, they show less positive findings than studies of the general population and also support some of the concerns raised above. In several studies, the administration of no/lo drinks prompted increased alcohol craving, autonomic arousal and increased the motivating properties of alcohol‐related cues. Plausible candidate mechanisms for this include associative learning, which might mean the presence of no/lo drinks can trigger alcohol cravings and, in turn, increase the risk of relapse to alcohol consumption [8, 31]. These conditioned responses may be particularly likely with contemporary no/lo drinks because these often have the same or similar branding and appearance as the more familiar higher‐strength products, have similar sensory properties (e.g. smell, taste), and are now more commonly consumed alongside other drinkers in environments such as pubs and restaurants. Alternatively, the small quantity of alcohol present in low‐alcohol drinks may produce a psychopharmacological ‘priming’ effect that triggers cravings and alcohol‐seeking behaviour [32, 33, 34]. Thus, for people with AUD who are attempting to abstain, exposure to or consumption of no/lo drinks may be a cue that increases the risk of relapse to heavy drinking [35, 36]. This applies to both alcohol‐free and low‐alcohol drinks, although the mechanisms may differ for these products and the risk for alcohol‐free drinks may be lower as these products may trigger lesser or no cravings when people know that their drink does not contain alcohol [37]. There may also be a particular risk for people with more severe AUD who are trying to maintain abstinence, and more evidence is needed to establish the relative risks and benefits of no/lo drinks in these contexts. Further, people who are required to abstain from alcohol as part of a court order or employment requirement (e.g. in mandatory sobriety schemes [38]) may be in breach of those requirements by consuming low‐alcohol products containing small quantities of alcohol.

Evidence relating to people with significant liver disease is even more sparse. Only two studies have examined the use of no/lo drinks in this group. First, a randomised controlled trial of patients with cirrhosis caused by metabolic dysfunction‐associated steatotic liver disease (MASLD) found that daily consumption of 330 ml of low‐alcohol beer was associated with improvements in nutritional status, endothelial function and quality of life, without evidence of harm [39]. Second, a retrospective cohort study identified a link between alcohol‐free beer consumption and increased rates of 6‐month abstinence in patients presenting for liver transplant assessment [40]. However, patients who consumed alcohol‐free beer at the time of diagnosis of liver disease were less likely to become completely abstinent. Overall, the evidence that no/lo products benefit or harm those with advanced liver disease is very limited.

## RESPONDING TO THE RISE OF NO/LO DRINKS

There is a need for more research, evidence and guidance on the use of no/lo drinks by higher‐risk groups.

For higher‐risk drinkers in the general population who are at risk of developing more severe AUD or ARLD, we need further and more robust evidence on whether and how these products can support reductions in alcohol consumption. This includes experimental and longitudinal observational studies that provide evidence on the extent to which no/lo drinks replace the consumption of standard alcoholic drinks and the mechanisms through which this occurs (e.g. prompting attempts to reduce consumption or improve the success of these attempts). Guidance is also required on whether and how to incorporate no/lo drinks within secondary prevention efforts targeting higher‐risk drinkers, such as brief interventions, social marketing, web‐based guidance and smartphone apps. Finally, studies should focus particularly on socio‐demographic groups with higher rates of alcohol‐related harm, including those of lower socio‐economic status and marginalised groups (e.g. sexual and gender minorities). Understanding whether and why higher‐risk drinkers in these groups are less likely to consume no/lo drinks and, if they have beneficial effects, how this might be overcome, may help to reduce alcohol‐related health inequalities.

For those with AUD, no/lo drinks are likely to have different risk and benefit profiles for different subgroups and, potentially, for alcohol‐free versus low‐alcohol drinks, although evidence on the details of both points is limited. Notwithstanding any increased risks, it is possible that attempting moderation by using no/lo drinks might still be beneficial overall, even for those with severe AUD, if only as an intermediate goal. For people with AUD who are already abstinent, no/lo products may present a risk of relapse, but these risks should be balanced against the potential benefits from increased opportunities for social interaction, integration and reduced experiences of stigma [41]. In line with current clinical guidelines on appropriate goals for drinking behaviour in the context of treatment for AUD, the advisability or otherwise of no/lo drinks (and the choice between alcohol‐free and low‐alcohol drinks) should be considered alongside other relevant factors in a negotiated care plan. However, if a patient’s choice is to use no/lo drinks with a moderation or controlled drinking goal, where the clinical advice is abstinence, they should not be denied treatment on this basis.

With regards to ARLD, major guidelines from the UK, Europe and the USA make no reference to no/lo drinks [42, 43, 44, 45, 46, 47, 48]. Our experience suggests that many clinicians adopt a zero‐tolerance policy towards these products, and a study of online advice from US doctors suggests mixed views on this [49]. Anecdotally, we have found that a zero‐tolerance approach can be confusing and unhelpful to patients who often do not believe no/lo drinks will cause them harm, particularly when compared with their previous drinking habits. Indeed, patients may feel proud of their success in reducing their alcohol intake and may be resistant to prohibitions on the product that has facilitated their achievement. A zero‐tolerance policy risks alienating patients and eroding their trust in clinicians, as may also be the case when treating AUD. Furthermore, zero‐tolerance approaches present a particular problem for the many patients with ARLD who do not self‐identify as having AUD [50], and who therefore do not recognise the risk of relapse posed by no/lo drinks. Guidance should ultimately take account of ARLD representing a spectrum of illness. A more liberal approach could be taken for patients at an earlier stage of disease, compared with those with decompensated cirrhosis or alcohol‐associated hepatitis. Conversely, no/lo drinks, and alcohol‐free drinks in particular, may be the lesser evil for patients with severe disease in whom reducing alcohol consumption is critical, but is unachievable by other means owing to the presence of severe AUD. Patients under consideration for liver transplant should also be aware of how the consumption of no/lo products, particularly low‐alcohol products, might affect their candidacy for transplant, and in particular that consumption of no/lo products can result in positive tests for urinary alcohol metabolites [51]. Finally, we should also consider patients with liver disease not caused by alcohol and what advice about no/lo drinks they should receive.

## CONCLUSION

The new generation of no/lo drinks may offer substantial benefits for people at risk of harm from heavy drinking and should therefore be considered as a potential tool in public health practice, treatment or recovery settings. However, these potential benefits are accompanied by potentially significant risks. Research is therefore needed to establish whether, how, and by whom no/lo drinks can be used effectively and safely to support reductions in high‐risk drinking, meeting specific treatment goals or achieving successful recovery. In particular we need multidisciplinary evidence from public health, psychiatry, addiction science, hepatology and social sciences to inform new interventions, as well as clinical and practitioner guidance in this area. This should extend across the full spectrum of prevention and treatment and be applicable to the populations at greatest risk of alcohol‐related harm, including lower socio‐economic or marginalised groups. For those with the most severe problems, including AUD and ARLD, evidence is needed to inform clinical advice on potential risks and benefits for patients with different needs and circumstances.

## AUTHOR CONTRIBUTIONS

**John Holmes:** Conceptualization (equal); writing—original draft (lead); writing—review and editing (lead). **Christopher K. Oldroyd:** Conceptualization (equal); writing—original draft (supporting); writing—review and editing (supporting). **Colin Drummond:** Conceptualization (supporting); writing—original draft (supporting); writing—review and editing (supporting). **Matt Field:** Writing—review and editing (supporting). **Inge Kersbergen:** Conceptualization (equal); writing—review and editing (supporting). **Michael E. D. Allison:** Conceptualization (equal); writing—review and editing (supporting).

## DECLARATION OF INTERESTS

J.H. and I.K. have received funding for ongoing, unrelated research on alcohol‐free and low‐alcohol drinks from Alcohol Change UK (ACUK), which received <0.6% of its funds in 2024–2025 from Lucky Saint, an organisation that produces and sells non‐alcoholic drinks and owns a pub that sells standard alcoholic drinks. In March 2025, Lucky Saint became an associate member of The Portman Group, a UK self‐regulatory organisation that is fully funded and controlled by the alcohol industry. ACUK has a strict policy of not accepting any funds from, nor being subject to any influence whatsoever from, the alcohol industry, including through its investment portfolio. ACUK has confirmed that it is in full compliance with this policy. At the time of publication, J.H. and I.K. were discussing their response to these developments.
